# Strain-based diffusion solver for realistic representation of diffusion front in physical reactions

**DOI:** 10.1371/journal.pone.0175695

**Published:** 2017-04-27

**Authors:** Jong-Hyun Kim, Jung Lee

**Affiliations:** 1 Department of Software Application, Kangnam University, Gyeonggi-do, Republic of Korea; 2 Department of Convergence Software, Hallym University, Gangwon-do, Republic of Korea; North China Electric Power University, CHINA

## Abstract

When simulating fluids, such as water or fire, interacting with solids, it is a challenging problem to represent details of diffusion front in physical reaction. Previous approaches commonly use isotropic or anisotropic diffusion to model the transport of a quantity through a medium or long interface. We have identified unrealistic monotonous patterns with previous approaches and therefore, propose to extend these approaches by integrating the deformation of the material with the diffusion process. Specifically, stretching deformation represented by strain is incorporated in a divergence-constrained diffusion model. A novel diffusion model is introduced to increase the global rate at which the solid acquires relevant quantities, such as heat or saturation. This ensures that the equations describing fluid flow are linked to the change of solid geometry, and also satisfy the divergence-free condition. Experiments show that our method produces convincing results.

## Introduction

Interactions between solids and fluids are very common in the real world, and many related phenomena have been modeled in computer graphics. Some scenarios, such as wetting clothes or burning paper, require the modeling of interacting phenomena with complicated characteristics including the deforming or tearing of the solid shapes. These phenomena include capillary action [1–3], friction [4], wetting [5, 6], fracturing [7], burning [8–10], and melting [11–13].

These phenomena can be modeled by allowing a model of water or fire as a fluid to permeate solid objects [1, 2, 8]. However, most techniques have suffered from representing detailed fluid flow across the surfaces of the solid during the diffusion process. An exception is the method proposed by Jeong et al. [8] to model the wrinkling and shrinkage of burning paper. They obtained very realistic crumpled surfaces, but unfortunately, monotonous patterns appear at the combustion front.

The simulation of the state change between a fluid and a solid involves modeling many complicated physical processes, such as heat transfer, as well as modeling the deformation of the solids. The occurrence of a monotonous pattern, especially during the diffusion step, produces isotropic propagation of physical quantities, such as heat or saturation, and makes it difficult to obtain a realistic simulation. We will show how monotonous wrinkling, tearing, and absorption patterns can be avoided by incorporating a strain term into a divergence-constrained diffusion equation.

To simulate the crumpling or tearing of objects by interacting phenomena such as burning or wetting, we need to analyze the flow of heat or fluid on their surfaces. When fire or water comes into contact with a solid, either burning or absorption causes various phenomena on the surfaces of solids. These phenomena include wrinkling, shrinkage, absorption, and saturation. Most previous techniques create artifacts, such as isotropic and monotonous patterns, in these regions; even anisotropic diffusion methods have some difficulty in expressing detailed phenomena, such as torn surfaces or dynamic absorption flow. Models of combustion can exhibit monotonous patterns on the flame front because they do not explicitly consider the deformation of the solid itself during the diffusion process. Although absorption-related methods have improved the realism of absorption flow using geometric information, monotonous patterns still appear and an awkward flow often happens in cases of large deformations, such as those that occur when flabby-like surfaces are agitated by moving water.

**Fig 1:**
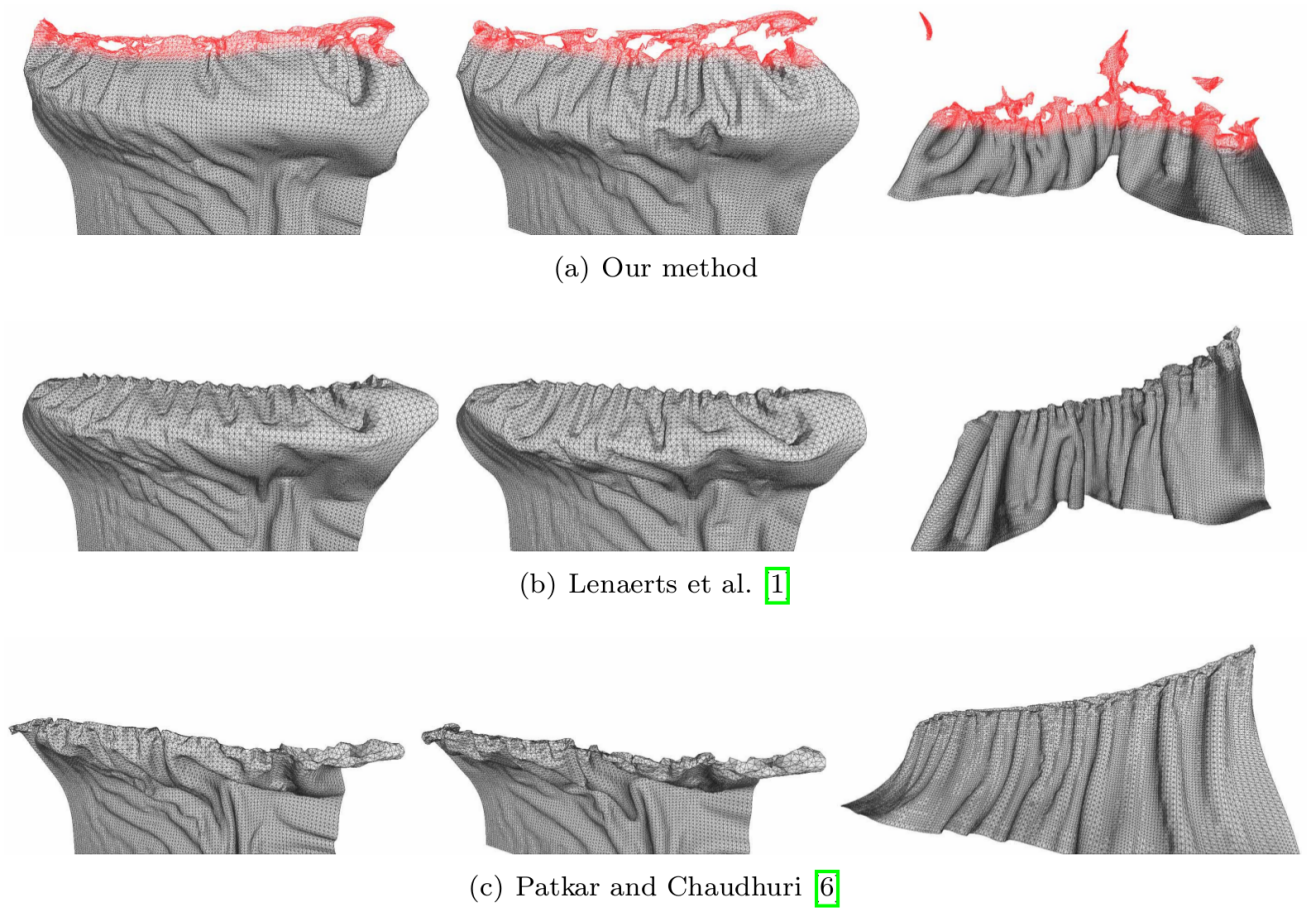
Quality of burning surfaces near the diffusion front. Comparison of burning surfaces by inserting various diffusion methods into the framework of Jeong et al. [8].

**Fig 2:**
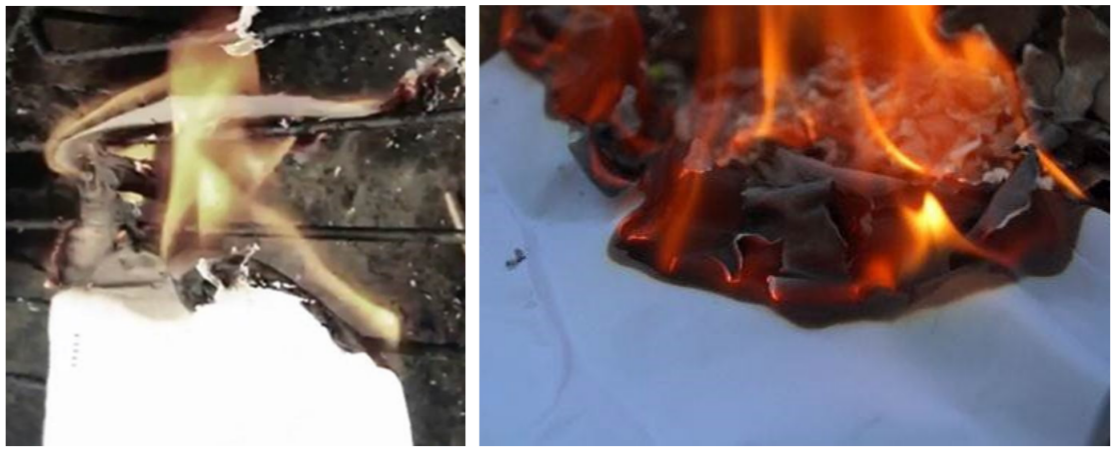
Real images of burning paper.

We observed the burning process before designing our framework. When cloth or paper is burning, deformation such as clumping and wrinkling occurs, leading to burning sheets on the burning front (see Figs 1 and 2). To express the burning process, a turbulence model based on texture or noise is commonly used in computer graphics. However, a predefined texture cannot represent a dynamic interaction such as clumping and wrinkling. Some anisotropic diffusion methods reduce this problem a little, but they are not enough to express dynamic features because they only consider directions of diffusion. In this paper, we will improve the details of diffusion during the burning process by analyzing strains from wrinkling or clumping (see Fig 1).

In a real world, diffusion speed usually increases if solid is deformed by external forces, and various phenomena appear. Some researches have expressed fluids released from porous materials due to the twisting or pressing forces [1], or wetting phenomenon controlling diffusion by considering diffusion angle and gravity [6]. In computer graphics, isotropic and anisotropic approaches are commonly used in various applications of computer graphics: isotropic diffusion based on Darcy’s law [1] or on heat equation [8–10, 12], directional angle based diffusion [6], surface reconstruction based on anisotropic kernel [14], and fluid sheets preservation [15, 16]. Recently, a method for expressing water absorption has been proposed by using an anisotropic diffusion according to the hair strand [2]. However, most of them have focused on controlling global movement and deformation. Actually, it is commonly observed that water propagation is accelerated when wet clothes are stretched. Besides, surface deformed by crumping, wrinkling, and shrinkage due to combustion makes its diffusion front more complicate.

In computer graphics, noise- or shrinkage-based methods are proposed to express phenomena occurring around diffusion front because there are no physics equation about these detailed turbulent effects. But they still have monotonous patterns and show some noisy artifacts. We introduce a geometric approach to capture details of diffusion front. A key idea is to constrain isotropically propagating physical quantities using strain of solid deformation and integrate this term into diffusion process. During this process, we improve the diffusion quality by proposing divergence-constrained solution of non-conserved physical quantities and conserving physical quantities.

## Related work

Fluid flow in porous material has been studied extensively in fluid mechanics. Lenaerts et al. [1] simulated this type of flow by approximating Darcy’s law [17], using a smoothed particle hydrodynamics (SPH) technique. Chen et al. [4] modeled the wetting of clothes using Lenaerts et al.’s method and together with a nonlinear model of friction. Huber et al. [5] improved Lenaerts et al.’s method by combining SPH with a finite element method. Patkar and Chaudhuri [6] proposed a new wetting framework by introducing the concept of diffuse gravity, in which the force caused by gravity diffuses the gradient derived from the geometry of the cloth. This reduces the isotropy and regularity of wetting patterns, but the flow of soaked water is still awkward because the absorption of water depends on the angle between the vertical and the diffused gradients.

You et al. [18] simulated the process of painting by combining the fluidity, diffusion, and absorption of pigment in water. Rungjiratananon et al. [3] and Lin [2] proposed an SPH-based method of coupling water and hair, which is based on the processes that take place in the wetting of hair, such as absorption and cohesion.

Losasso et al. [12] modeled burning and melting effects using a body-centered cubic lattice. Melek et al. [10] expressed the crumpling of burning solids using free-form deformation (FFD). Liu et al. [9] modified Melek et al.’s method and modeled the deformation of burning thin shells. These FFD-based methods produce unrealistic wrinkling and shrinkage of burnt areas because they are based on a reduced coordinate system.

Larboulette et al. [19] modeled a thin shell object which acts as the mass spring system. In their approach, the vertices of the surface mesh become the mass points and their connecting edges become the springs, which represent the paper’s fiber. The mass spring system is coupled to a heat propagation solver, which consists of particles transferring energy onto the mass nodes. A change in heat on those nodes affects the length of the connected springs, which are cut or shortened in response. However, their method still have some monotonous patterns in expressing crumpling effects around diffusion front.

Jeong et al. [8] addressed this problem in modeling the shrinkage and wrinkling surfaces of clothes or paper by adjusting the rest length of springs connecting particles as the mass of these particles changed.

Methods mentioned earlier have focused on solid-fluid interactions such as shape of diffusion and deformation of solids. But isotropic diffusion reduces the quality of visual reality by showing monotone patterns in spite of solid deformation such as shrinkage or wrinkling. We enhance details of diffusion results by incorporating solid deformation into diffusion process.

## Strain-based divergence-constrained diffusion

Actual wetting and burning effects have many subtle features: for example, nonmonotony diffusion around the front of active regions. To express the change of features occurring when solids are deformed by fire or water, we propose a method that enhances the details of the front at the diffusion stage.

### Constrained diffusion

When a solid is wetted or burnt, it absorbs physical quantities. In this paper, we modified the method of Jeong et al. [8] to diffuse the absorbed physical quantities. First, we will review their method and then explain how to integrate it into our method.

After time Δ*t* expires, a physical quantity diffused on the surface is represented as follows:

$$\begin{array}{c} u\left(t+\Delta t\right)=u\left(t\right)+C\left(t\right)+u_{\text{ext}}\left(t\right), \end{array}$$ (1)

where **u** is a physical quantity such as water or heat that will be diffused on a surface, **u**_ext_ denotes the influx of physical quantities from water or heat sources, and *C* is a strain-based geometric constraint. Using this mechanism, we can control the physical quantities on surfaces. Eq (2) simulates absorption of water or burning through physically based diffusion.

$$\begin{array}{c} \frac{\partial u}{\partial t}-\alpha {▿}^{2}u=0, \end{array}$$ (2)

where *α* is thermal diffusivity.

To solve this equation for our particle-spring structure, we use Desbrun et al.’s method [20], which approximates an umbrella operator to the Laplacian. As the spring lengths in our model are not identical and are changed by deformation, we need to consider them as parameters in the diffusion equation. We use a scale-dependent umbrella operator that weights the Laplacian as the inverse of the distance between vertices:

$$\begin{array}{c} L\left(x_{i}\right)=\frac{2}{E}\sum_{j\in N_{1}\left(i\right)}\frac{x_{j}-x_{i}}{|d_{ij}|},\;\text{where}\;E=\sum_{j\in N_{1}\left(i\right)}\left|d_{ij}\right|, \end{array}$$ (3)

where **x** is the position of a vertex, *d*_*ij*_ is the distance between two vertices *v*_*i*_ and *v*_*j*_, and *N*_1_(*i*) is a 1-ring neighbor of a vertex *i*. This enables the uniform transfer of heat or water across irregular meshes. We can have longer time steps and higher thermal diffusivity, *α*, in a stable simulation of burning or wetting using the implicit backward Euler method.

### Geometric constraints

**Fig 3:**
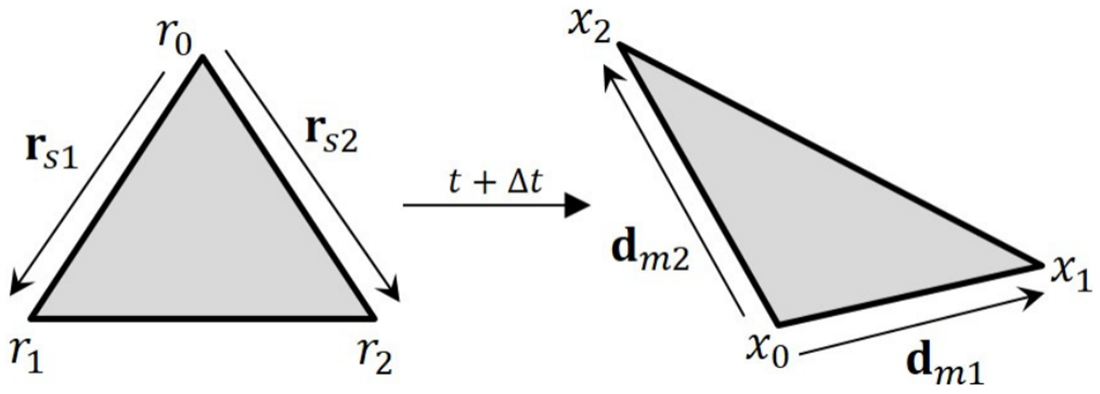
Edge vectors after deformation.

Our technique is based on strain, which we compute as follows: Given three vertices **x**_0_, **x**_1_, and **x**_2_ of a triangle and their reference configurations **r**_0_, **r**_1_, and **r**_2_ in 3D space, we compute the edge vectors between before and after deformation as **d**_*m*1_ = **x**_1_ − **x**_0_, **d**_*m*2_ = **x**_2_ − **x**_0_, **r**_*s*1_ = **r**_1_ − **r**_0_, and **r**_*s*2_ = **r**_2_ − **r**_0_. We construct matrices **D**_*m*_ with columns **d**_*m*1_ and **d**_*m*2_, and **D**_*s*_ with columns **r**_*s*1_ and **r**_*s*2_ (see Fig 3).

For each triangle, we can then obtain the deformation gradient $F=D_{m}D_{s}^{-1}$, and hence, the Green strain tensor **G** = (**F**^*T*^**F** − **I**)/2. A geometric constraint *C* can now be computed:

$$\begin{array}{c} C=\frac{1}{2}\cdot A\cdot S \end{array}$$ (4)

where *A* is the area of the triangle, and where *S* is its stretch which is computed as follows [21, 22].

$$\begin{array}{ccc} S & = & \lambda G{\left(0,0\right)}^{2}+\lambda G{\left(1,1\right)}^{2}+2\lambda G\left(0,0\right)G\left(1,1\right)\\ & & +\lambda G{\left(0,1\right)}^{2} \end{array}$$ (5)

The constant λ is set to $\frac{1}{1+\delta_{\text{weakening}}}$, where *δ*_weakening_ is a user-defined threshold. *δ*_weakening_ in the above equation denotes force relaxing the strength of stretching force. If it becomes larger, surfaces tend to maintain equilibrium stronger, and this results in weakening influence of stretching force.

**Fig 4:**
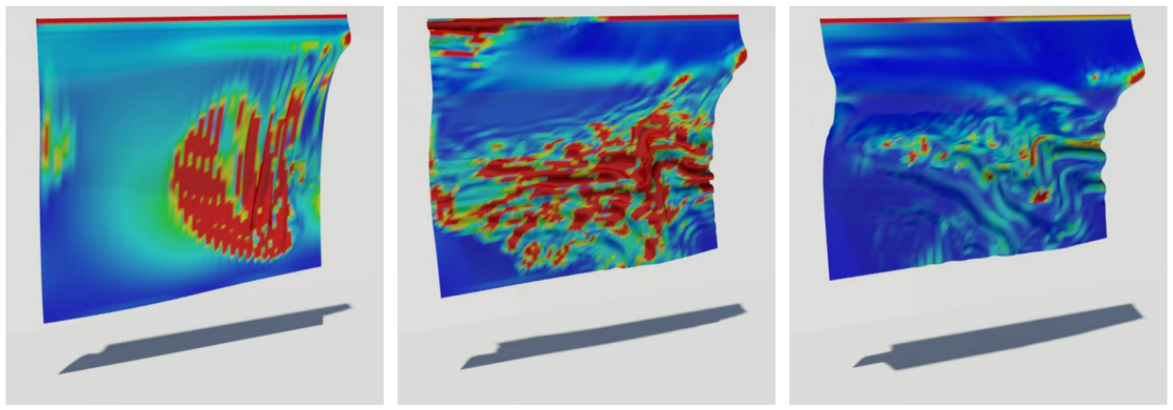
Geometric constraint. Values of geometric constraint *C* produced by an external force as time passes. The magnitude of the constraint is represented with color spectrum, from low (blue) to high (red).

**Fig 5:**
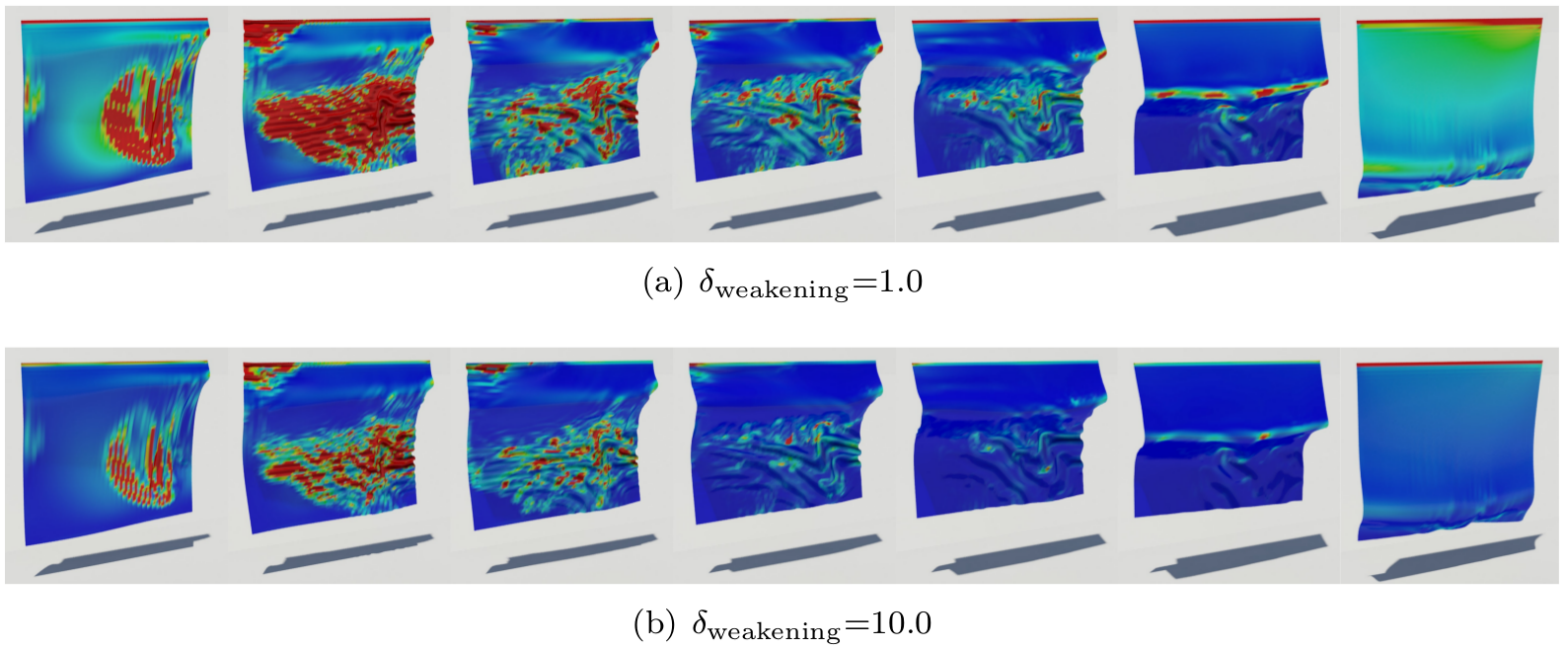
Different weakening thresholds.

Fig 4 shows values of the *C* during the deformation of an object and the results of wrinkling and shrinkage very well. We then solve a divergence-constrained diffusion equation with this constraint. Fig 5 compares the results diffused by different weakening values that indicate how much the strains are relaxed. Using this value, we can produce various scenes by adjusting strength of strain affecting the diffusion process. *δ*_weakening_ value is an experimentally adjusted constant through several experiments. As shown in Fig 5, the user can adjust this value to express various scenes. We have further inserted in the paper on what results are generated when this value is changed. If this value is increased, the diffusion pattern of physical reactions such as wetting and burning is strongly affected by external force (see Fig 5b). This controllability can lead to a diffuse pattern in which various solid materials are considered during burning simulation. When this value is increased, the cooling effects of diffused temperature are more strongly expressed in the burning simulation. (cooling effects: It is a phenomenon that the temperature is lowered when burning. In general, when simulating fire or burning, a term is added to cool the temperature.)

### Divergence-constrained diffusion

Some researchers [23, 24] have suggested generating a fluid-like motion in an Eulerian framework without solving the Poisson equation. Instead, they create a divergence-constrained vector field **u**_**vel**_ using a moving least-squares fitting process. Similarly, we try to improve the solver by constraining the divergence. Huerta et al. [25] used a divergence constraint,

$$\begin{array}{c} ▿\cdot u_{\text{vel}}=\sigma , \end{array}$$ (6)

to improve the description of subgrid incompressibility with *σ* = 0. **u**_**vel**_ in the above equation denotes velocity field, and we model diffusion processing as follows by using the concept of divergence-constrained solver.

$$\begin{array}{c} \frac{\partial u^{*+C}}{\partial t}-\alpha {▿}^{2}u^{*+C}=0. \end{array}$$ (7)

The above equation is a diffusion equation improved from Eq (2) by considering strain, where **u**^∗+*C*^ denotes physical quantity with constraint *C*.

*C* denotes a strain value that is shrunk and wrinkled on the surface of a solid due to burning or wetting simulation. This value is measured more strongly in the area where deformation such as shrinkage, wrinkling, and ablation occurs. Because the deformation of the solid affects the physical reaction, we have added strain-based *C* to **u** to improve the detail of the diffusion front. Thus, **u** is not only spreading more dynamically due to the strain value *C*, but also improving the detail of the diffusion front.

**Fig 6:**
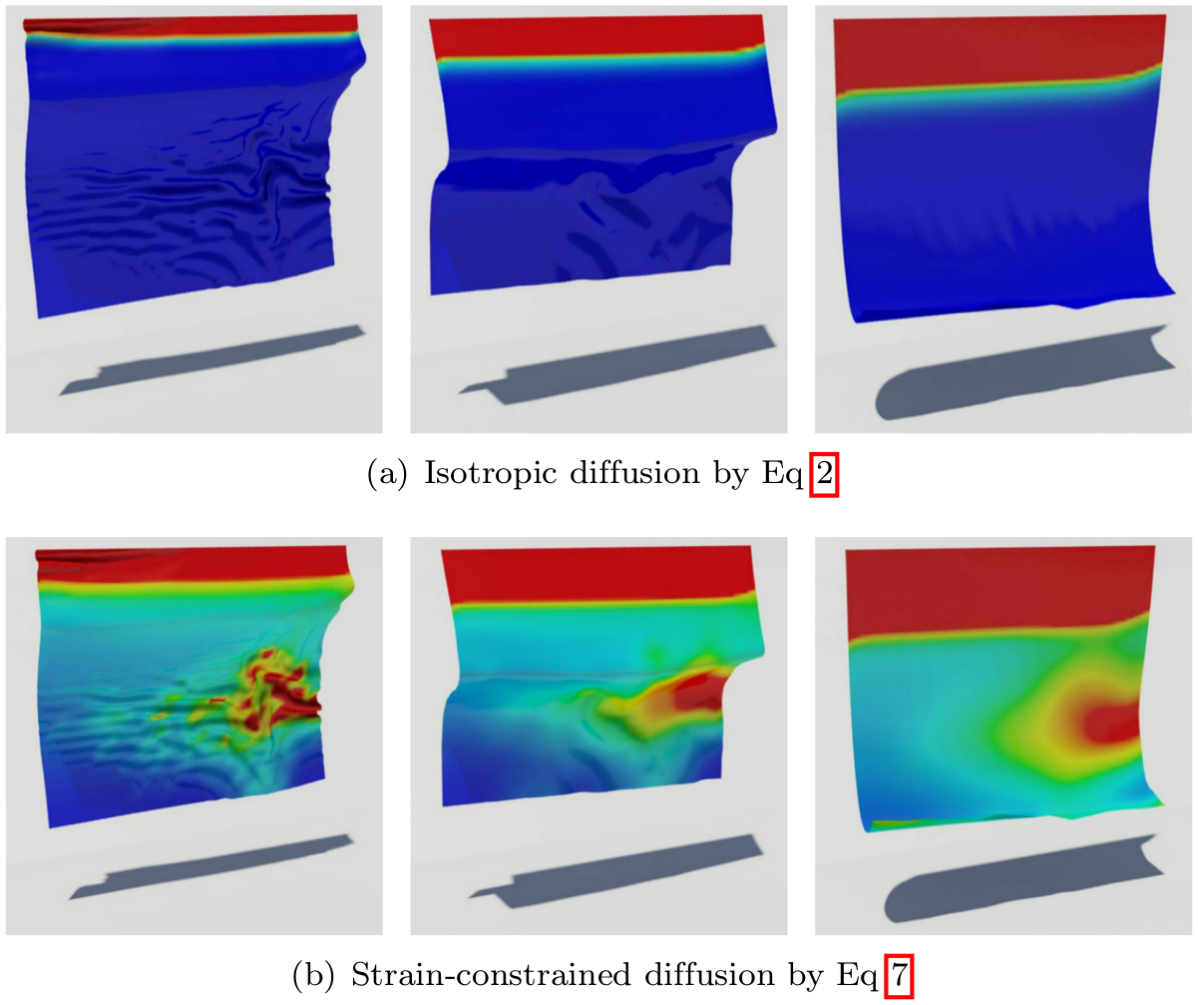
Comparison of diffusion equations.

Eq (7) with constraints suppresses isotropic patterns, but still might cause unexpected artifacts to appear in regions away from the diffusion due to the inconsistency between the deformation by diffusion and geometric strain. These artifacts result in an awkward simulation (see Fig 6b). Artifacts appear when diffusion is modeled by Eq (7) alone. The strain by a large deformation in the middle of the object causes the diffusion to occur in the area where no physical quantities are transferred.

There appears an artifact increasing physical quantities if we simply add **u** and *C* because there are no explicit connection between them. To solve this problem, we find diffusion front areas using **u**_*min*_ and **u**_*max*_ and add a constraint *C* to **u** only in these areas (see Eq (8c)). As a result, we add physical quantities in diffusion front areas to strain, and improve the details of diffusion front areas by re-distributing these quantities with divergence-constrained condition using Eq (8). A condition for finding diffusion front is **u**_*min*_ ≤ **u** ≤ **u**_*max*_, where **u**_*min*_ and **u**_*max*_ are user-defined thresholds to break monotonous patterns.

$$\begin{array}{c} u^{*}\;\leftarrow \;solve\;\frac{\partial u}{\partial t}-\alpha {▿}^{2}u=0 \end{array}$$ (8a)

$$\begin{array}{c} u^{*,sum}=\sum_{i}^{n}u_{i}^{*} \end{array}$$ (8b)

$$\begin{array}{c} u^{*+C}=u^{*}+C \end{array}$$ (8c)

$$\begin{array}{c} \sigma^{target}=u^{*,sum}\sigma \end{array}$$ (8d)

$$\begin{array}{c} u_{i}^{*,scale}=\frac{u_{i}^{*+C}}{\text{max}(u_{0}^{*+C},...,u_{n-1}^{*+C})} \end{array}$$ (8e)

$$\begin{array}{c} u^{*,sumScale}=\sum_{i}^{n}u_{i}^{*,scale} \end{array}$$ (8f)

$$\begin{array}{c} u\;\leftarrow \;u^{*,target}=\frac{u^{*,scale}}{u^{*,sumScale}}\sigma^{target}, \end{array}$$ (8g)

where *n* is the total number of vertices in the mesh, and Eq (8a) computes u* using Eq (6) before applying the constrained diffusion condition. In Eq (8b), **u**^∗,*sum*^ denotes the sum of physical quantities of entire vertices and is used to control the amount of physical quantities changed by strain. **u**^∗+*C*^ in Eq (8c) involves an error of increasing physical quantities due to the addition of geometric constraints. The physical quantities changed by constraint *C* in Eq (8c) are adjusted to be divergence-free by Eqs (8d)–(8g), where *σ* is another user-defined threshold, which controls the divergence. If *σ* = 1, *σ*^*target*^ becomes **u**_∗,*sum*_, and the divergence-free condition is satisfied.

**Fig 7:**
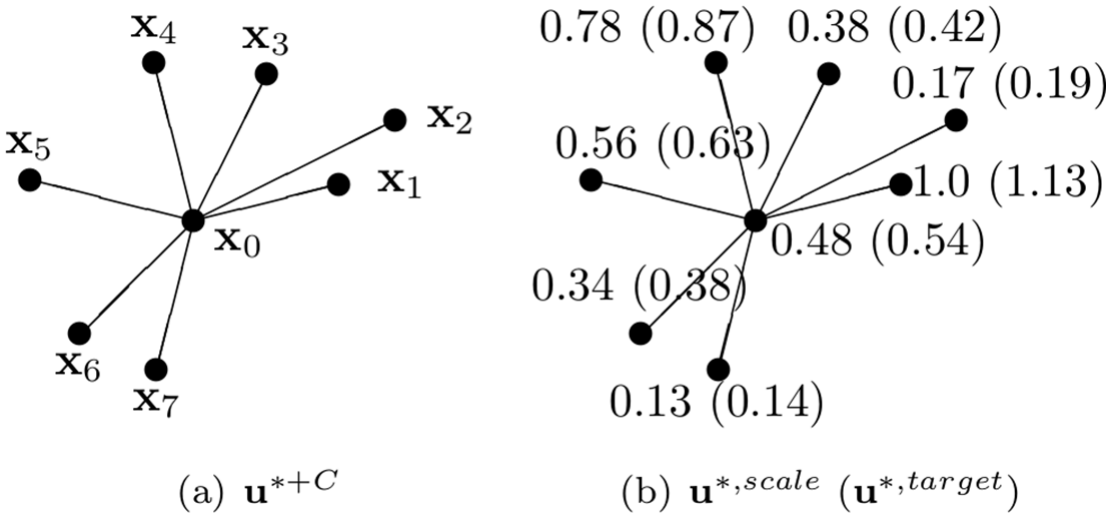
Example. Divergence-constrained condition with *σ*^*target*^ = 4.3. (a) A set of **u**^∗+*C*^ = {5.7, 12.0, 2.0, 4.5, 9.3, 6.7, 4.1, 1.5}, (b) values of **u**^∗,*scale*^ and **u**^∗,*target*^ for **x**_0,1,..,7_.

The term *σ*^*target*^ in Eq (8d) applies a constraint to the diffusion front and details follow hyperbolic patterns. Values of the divergence-constrained condition in a simple example are shown in Fig 7.

1. Fig 7 assumes the physical quantities increased by adding the constraint *C*.
2. **u**^∗,*scale*^ is normalized to the maximum value **x**_1_ = 12. Eq (8e) is evaluated with the results shown in Fig 7b.
3. The value of **u**^∗,*sumScale*^ is 3.82 from Eq (8e), and **u**^∗,*scale*^ is rescaled by this value to determine **u**^∗,*target*^ (see Eq (8g)). The total physical quantity of the rescaled **u**^∗,*target*^, $\sum_{i}^{n}u_{i}^{*,target}=4.3$, which equals to *σ*^*target*^. Therefore, the momentum is preserved to the extent specified by the user.

Our technique can model anisotropic diffusion. Because it takes the deformation of solids into account, it is also possible to model hyperbolic diffusion through the surface. Table 1 shows the values computed by Eqs (8c)–(8g) for Fig 7. In this table, we can see that the entire physical quantity *σ*^*target*^ is the same as the refined sum of the physical quantities. Using this mechanism, we can easily control the user-defined hyperbolic diffusion as well as the divergence-free condition. We solve *C* based on triangles, re-distribute it by a third with vertex level, and solve Eq (8)*.

**Table 1:** Values for Fig 7.

Index	u^∗+C^	*σ*	*σ*^*target*^	Eq (8e)	Eq (8f)	Eq (8g)
0	5.7	1.0	4.3	0.48	3.82	0.54
1	12.0	–	–	1.0	–	1.13
2	2.0	–	–	0.17	–	0.19
3	4.5	–	–	0.38	–	0.42
4	9.3	–	–	0.78	–	0.87
5	6.7	–	–	0.56	–	0.63
6	4.1	–	–	0.34	–	0.38
7	1.5	–	–	0.13	–	0.14

In this paper, **u** is the physical quantity of a material, such as water or heat, that seeps into the surface of a solid, and determines the amount that it seeps and diffuses into the solid surface when it collides with a solid. *C* denotes a strain value that is shrunk and wrinkled on the surface of a solid due to burning or wetting simulation. This value is measured more strongly in the area where deformation such as shrinkage, wrinkling, and ablation occurs. Because the deformation of the solid affects the physical reaction, we have added strain-based *C* to **u** to improve the detail of the diffusion front. Thus, **u** is not only spreading more dynamically due to the strain value *C*, but also improving the detail of the diffusion front. We propose a way to enforce the increased physical quantity divergence-free in this process (see Eq (8)*). As mentioned earlier, **u** and *C* do not improve the physical accuracy, but we propose a way to visually-convince the details of the diffusion front as it appears in real phenomena.

## Implementation

All our tests were performed on an Intel i7-2600k 3.40GHz CPU with 16GB of RAM and an NVIDIA GeForce GTX 580 graphics card. We used Autodesk 3ds Max to render the simulation results. All the solids were deformed using position-based dynamics [26], and smoke was simulated using Stable Fluids [27].

In Section ***Burning of a sheet material***, the density of smoke is determined by the mass lost in the burnt regions and the interaction between smoke and cloth is implemented using the method of Chentanez et al. [28]. Results of Sections ***Water absorption*** and ***Drooping surfacess*** focus on the water absorption, and friction force or water-dripping effects are not applied. We use the method of Ando and Tsuruno [29] as a water solver shown in the scene of water absorption, and the method of Batty et al. [30] for water–solid coupling.

## Results

We experimented with the following three scenarios. To show the robustness of our method, we tested three different scenes: (a) the shredding of a combustible sheet such as cloth as it burns; (b) the absorption of water by porous material; and (c) the absorption of water by a sheet material such as cloth, which droops as it becomes flabbier. In (a), the burning sheet is remeshed using marching triangles [12].

### Burning of a sheet material

**Fig 8:**
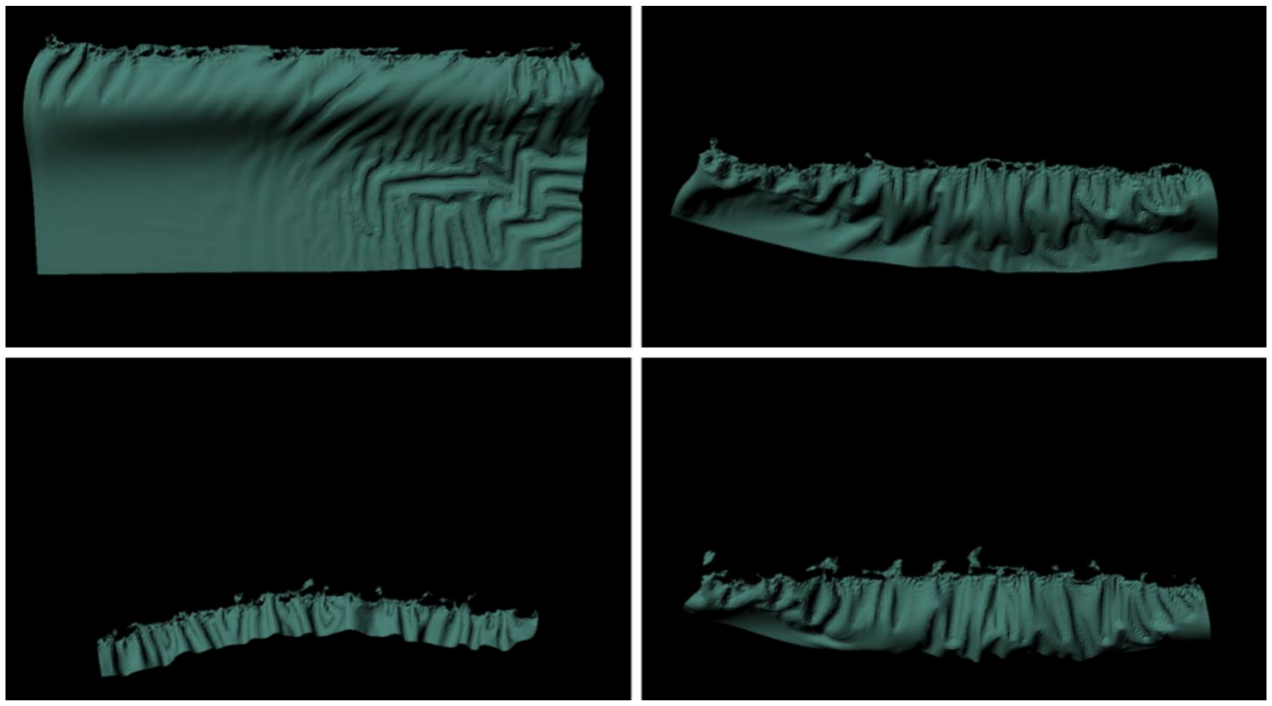
Sheet of paper. A sheet of paper is shredded during combustion, implemented by our method (*σ* = 1.0).

To model the shredding process, we inserted our model of the heat diffusion process into Jeong et al.’s algorithm, which models shrinkage, wrinkling, and ablation effects [8]. Fig 8 shows stages in the burning of a piece of cloth, modeled by our method. Shredding is clearly taking place around the burning front.

**Fig 9:**
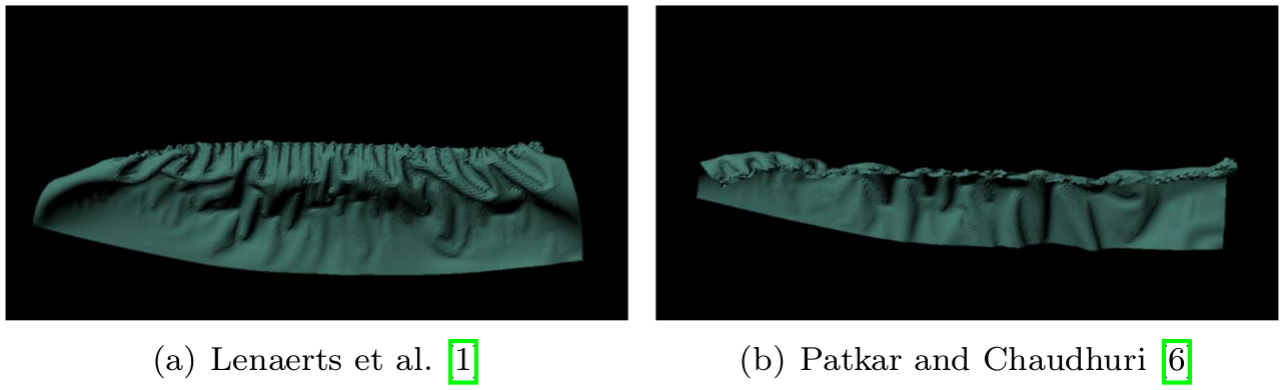
Sheet of paper with previous method. Models of burning sheets produced by previous methods.

When a similar scenario is modeled by the technique of Lenaerts et al. [1] or Patkar and Chaudhuri [6], we see monotonous patterns around the combustion front (see Fig 9). Jeong et al.’s method also produces isotropic patterns because their diffusion method is similar to that of Lenaerts et al.

**Fig 10:**
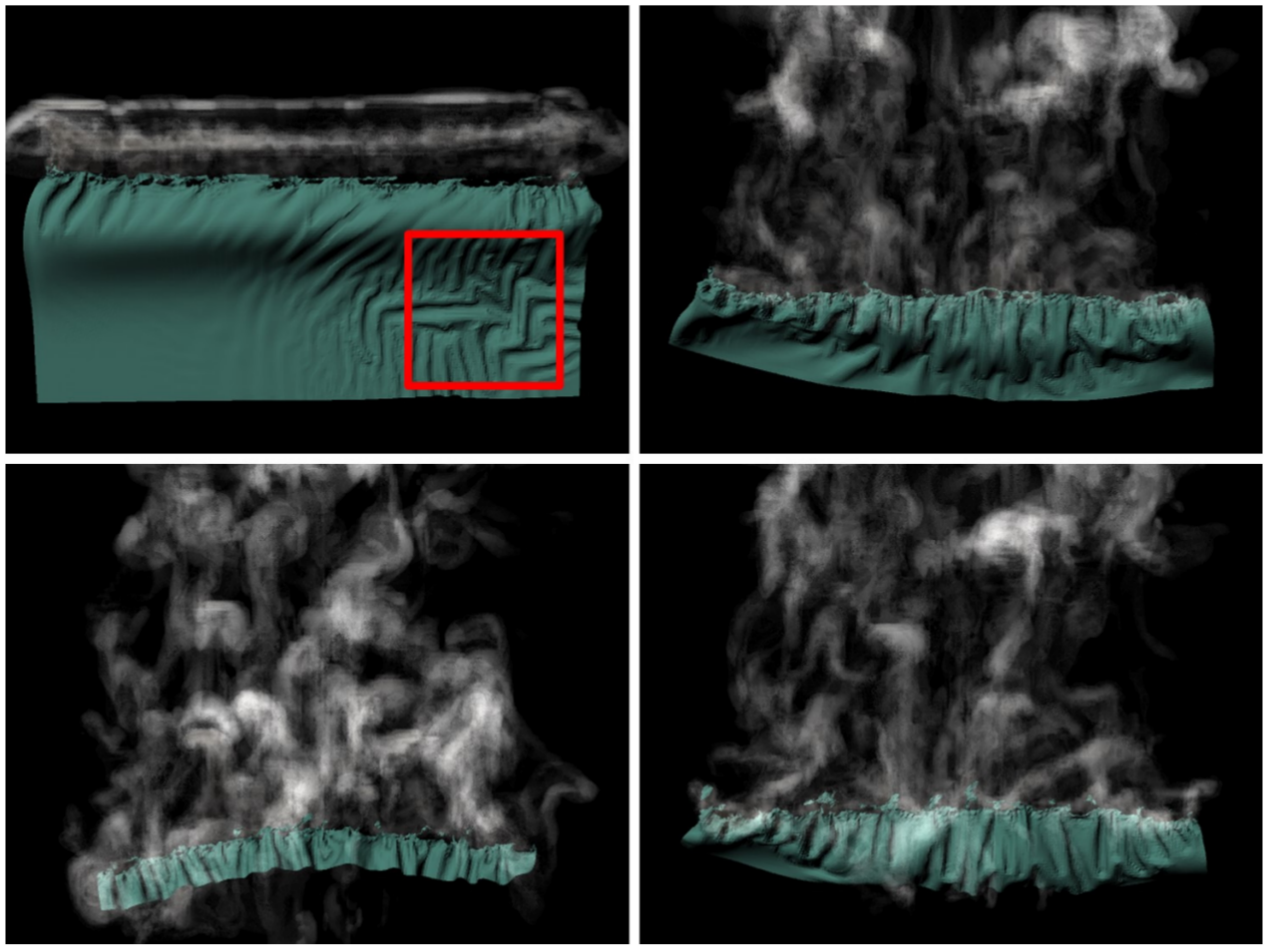
Sheet of paper and smoke. Simulation of a sheet that shreds as it burns, producing turbulent smoke, modeled by our method. The resolution of the grid for modeling the smoke is 128 × 128 × 128.

The complicated velocity field from torn edges around the combustion front causes the smoke to exhibit turbulent advection. In the red box of Fig 10, some wrinkles are observed away from diffusion front appear. They are results deformed by the upward-stretching force, not the artifacts by our method. We improve the details of burning sheets without any loss of wrinkling or shrinkage of underlying materials because monotonous patterns are broken around the diffusion front selected by the thresholds **u**_*min*_ and **u**_*max*_.

### Water absorption

**Fig 11:**
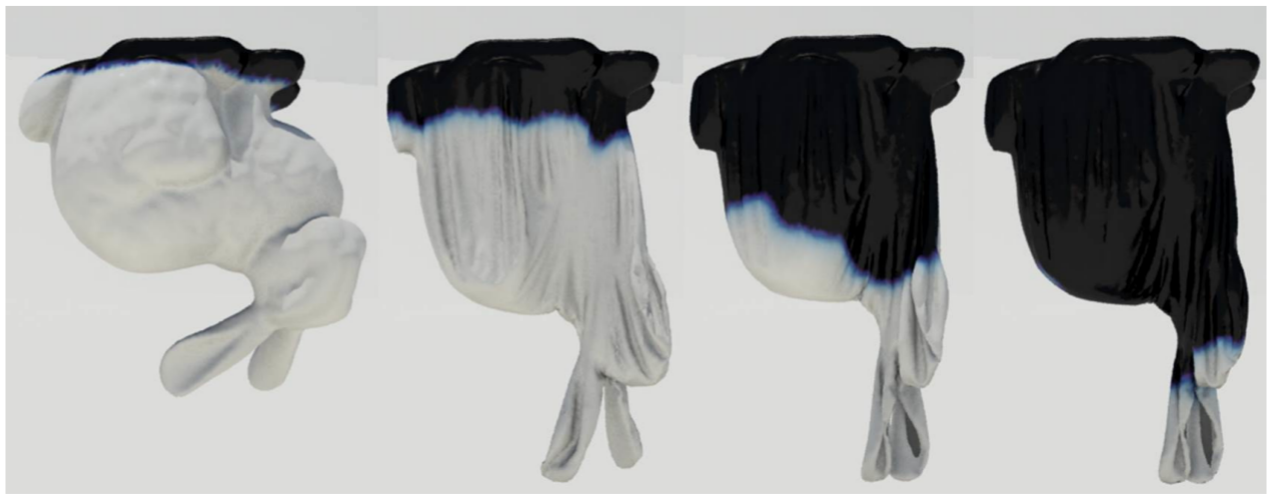
Deformable bunny absorbing water. Water absorption by a thin sheet, in the shape of the surface of the Stanford Bunny, modeled by our method (*σ* = 1.0).

**Fig 12:**
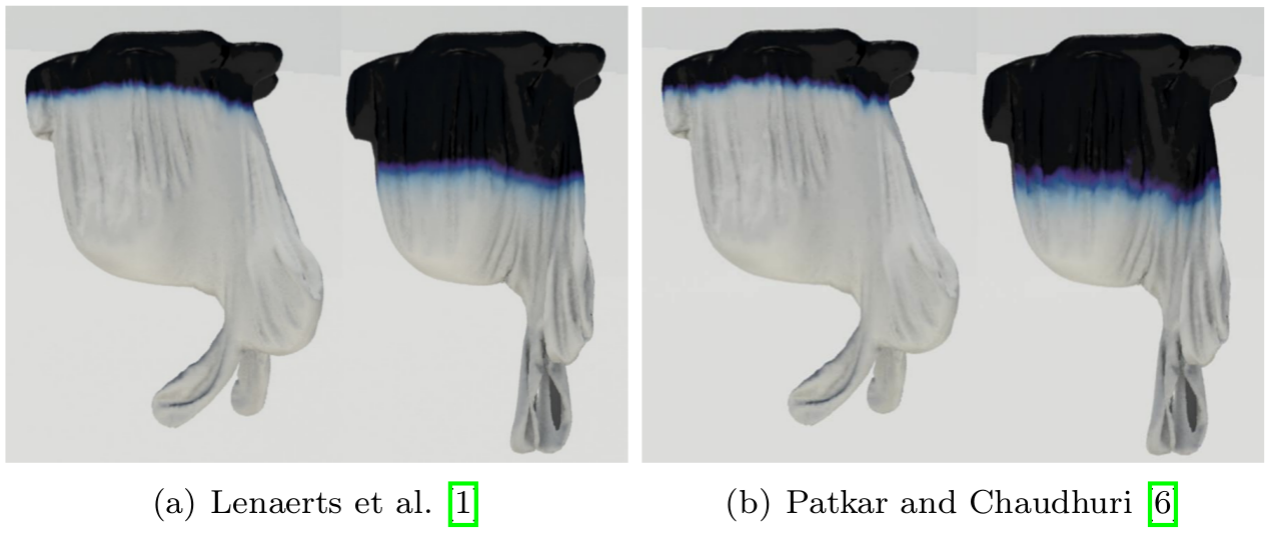
Deformable bunny absorbing water with previous methods. Water absorption by a thin sheet, modeled by previous methods.

Figs 11 and 12 compare patterns of water absorption by thin surfaces produced by our method and previous techniques. Our method produces anisotropic absorption patterns affected by the deformation of the object, whereas the isotropic diffusion produced by previous techniques seems to be irrelevant to the object deformation. Large deformation of solids is a very important factor of accelerating diffusion, and Lenaerts et al.’s method [1] also shows the acceleration, but isotropically. In contrast to their method, our method shows more active diffusion results because strain calculated per triangle affects the diffusion process.

**Fig 13:**
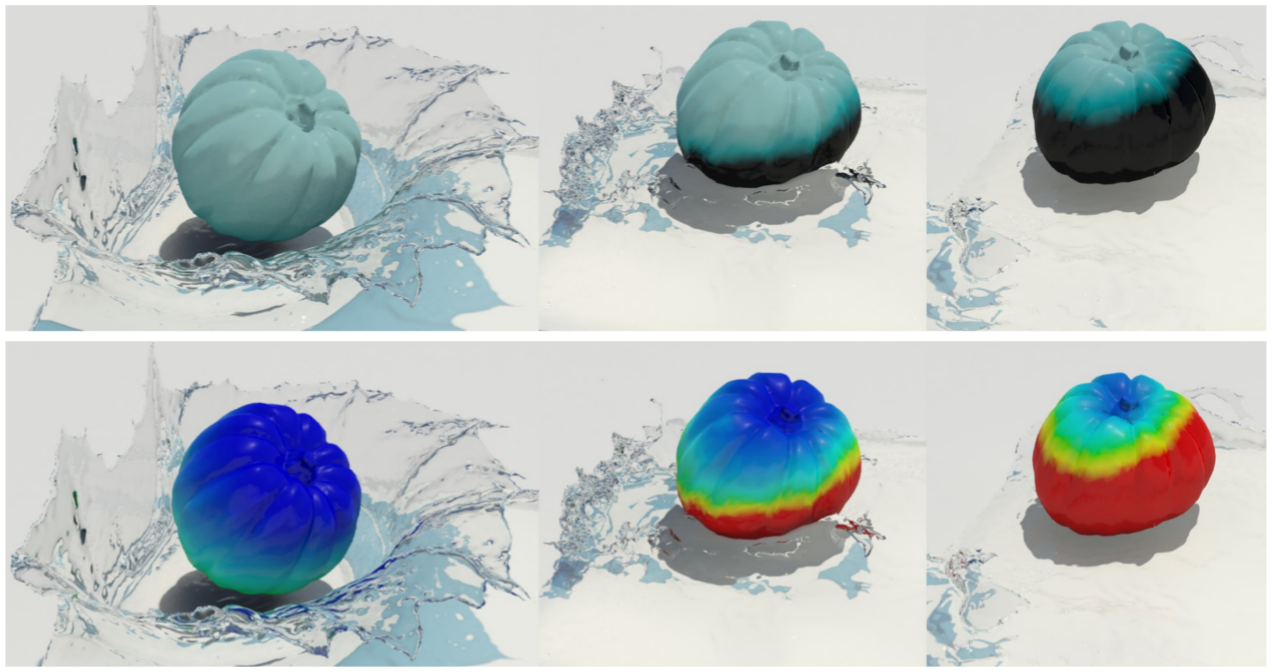
Deformable pumpkin absorbing water (*σ* = 1.0). The images in the left column show the absorption patterns, while those on the right show the extent of water absorption (red: wet, blue: dry).

Fig 13 shows water absorption of a solid, modeled using the method of Ando and Tsuruno [29]. In this scenario, absorption is controlled simply by checking the remaining capacity of the solid to determine whether additional water can be absorbed or not. Our method again produces anisotropic absorption patterns plausibly related to the deformation of a solid.

### Drooping surfaces

**Fig 14:**
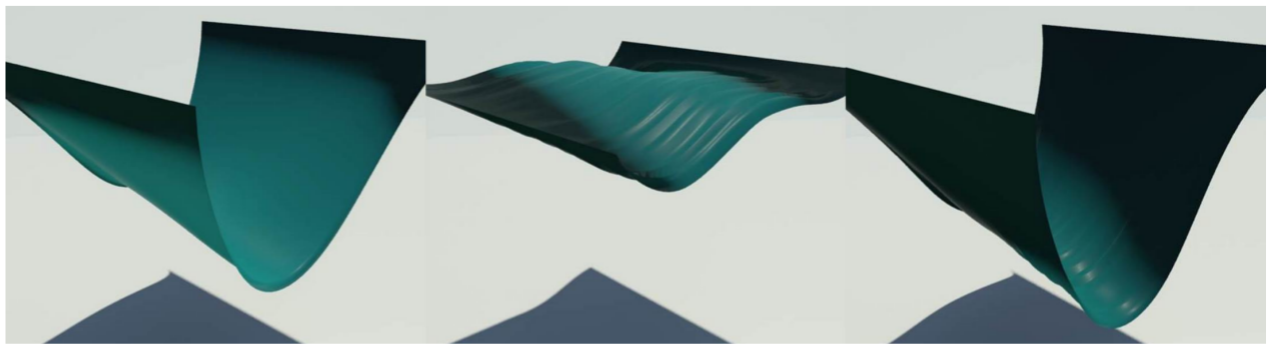
Drooping cloth. A cloth droops as it absorbs water, modeled by our method (*σ* = 1.0).

We inserted our algorithm into the method of Chen et al. [4] for modeling surfaces that droop due to water absorption. Fig 14 shows a scenario in which water is injected from each end of a piece of cloth, which absorbs to an extent that depends on the strain.

**Fig 15:**
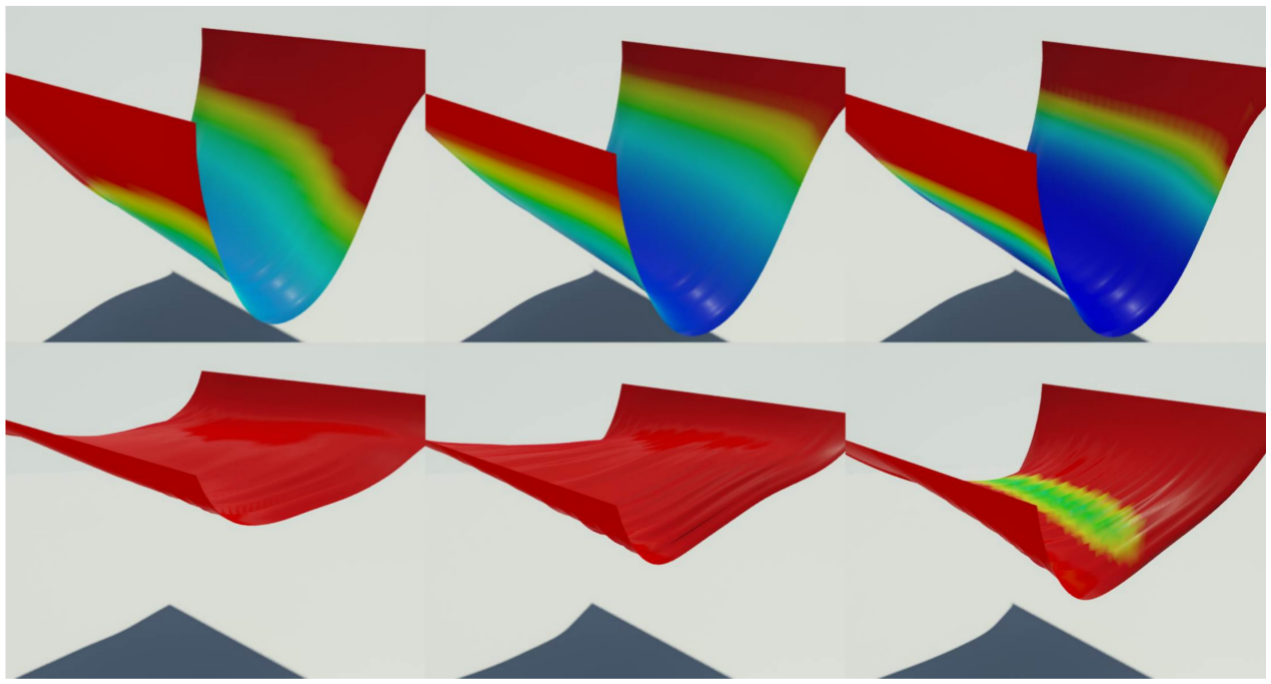
Comparison of drooping cloth. A cloth droops as it absorbs water, modeled by different methods (left column: our method, middle column: Lenaerts et al. [1], right column: Patkar and Chaudhuri [6]).

Fig 15 compares the pattern of water absorption produced by our method with previous techniques. Our result shows the stretching of the cloth affecting water absorption, while the techniques of Lenaerts et al. [1] or Patkar and Chaudhuri [6] produce monotonous patterns of absorption that are unrelated to the deformation. In addition, Patkar and Chaudhuri’s technique has difficulty in modeling diffusion around flat surfaces and ridges.

**Fig 16:**
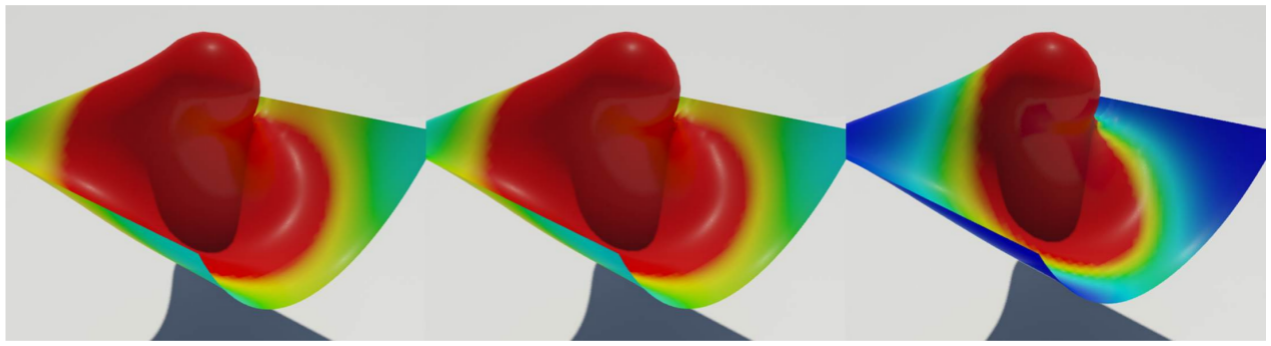
Stretched cloth. A stretched cloth is struck by a ball of water, modeled by different methods (left column: our method, middle column: Lenaerts et al. [1], right column: Patkar and Chaudhuri [6]).

Fig 16 shows another scenario, in which a square piece of cloth is struck by a ball of water with sufficient force so that the deformation of the cloth caused by the impact determines the pattern of water absorption. This figure compares the results in the same scenario, modeled using our method and the techniques of Lenaerts et al. [1], and Patkar and Chaudhuri [6]. Again, the last of these techniques produces insufficient diffusion.

### Divergence control

**Fig 17:**
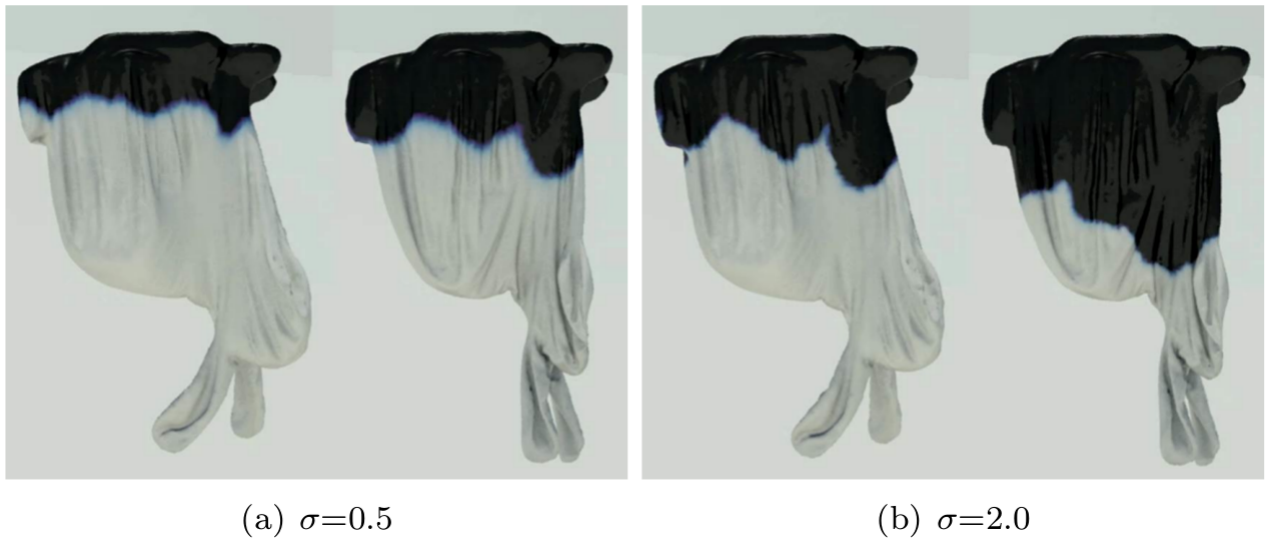
Controlling water absorption. Controlling the rate of absorption by adjusting *σ*.

Kim et al. [31] adjust divergence to control the volume of air bubbles, and Feldman et al. [32] uses it to control explosion effects. We can exaggerate the extent of diffusion by adjusting divergence. Fig 17 shows that a high *σ* value makes the diffusion fast while the detailed patterns are preserved.

**Fig 18:**
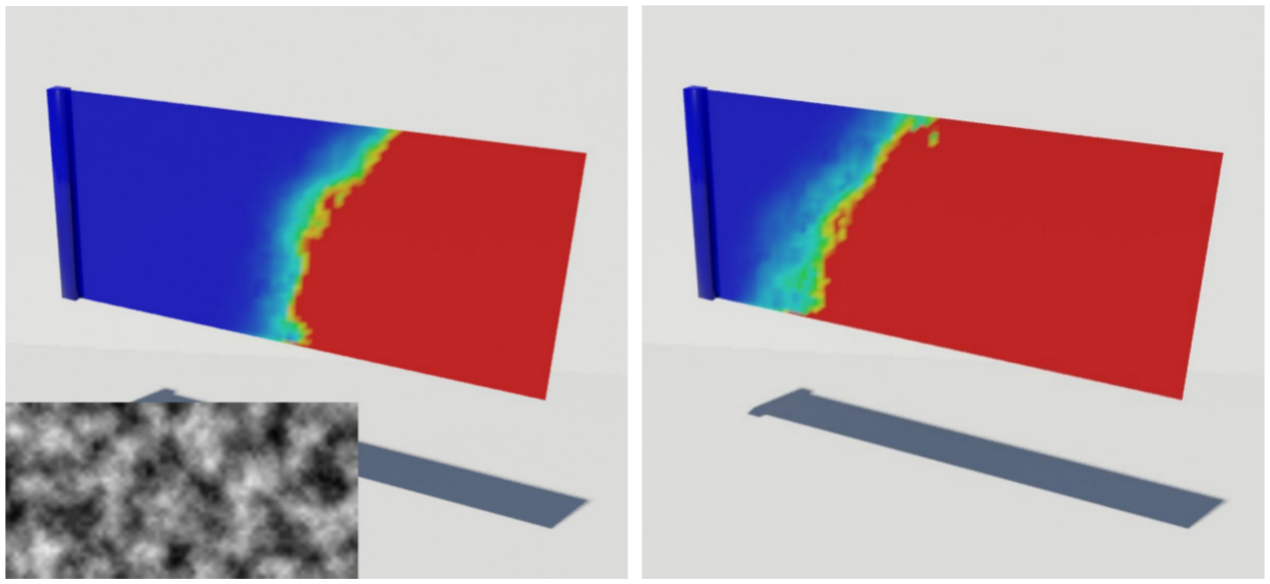
Controlling diffusion front with texture. Results controlled by applying noise texture to our method.

Fig 18 shows the results controlled by our method with Perlin noise texture. Diffusion actively advances according to the solid deformation and details of diffusion front is controlled by noise texture, as well. If we use only noise texture, monotonous patterns of diffusion front might be avoided temporarily but diffusion will be processed without taking into consideration of solid deformation. As seen in the figure, active diffusion can be maintained and details of diffusion front can be easily controlled if noise texture is integrated into our method.

**Fig 19:**
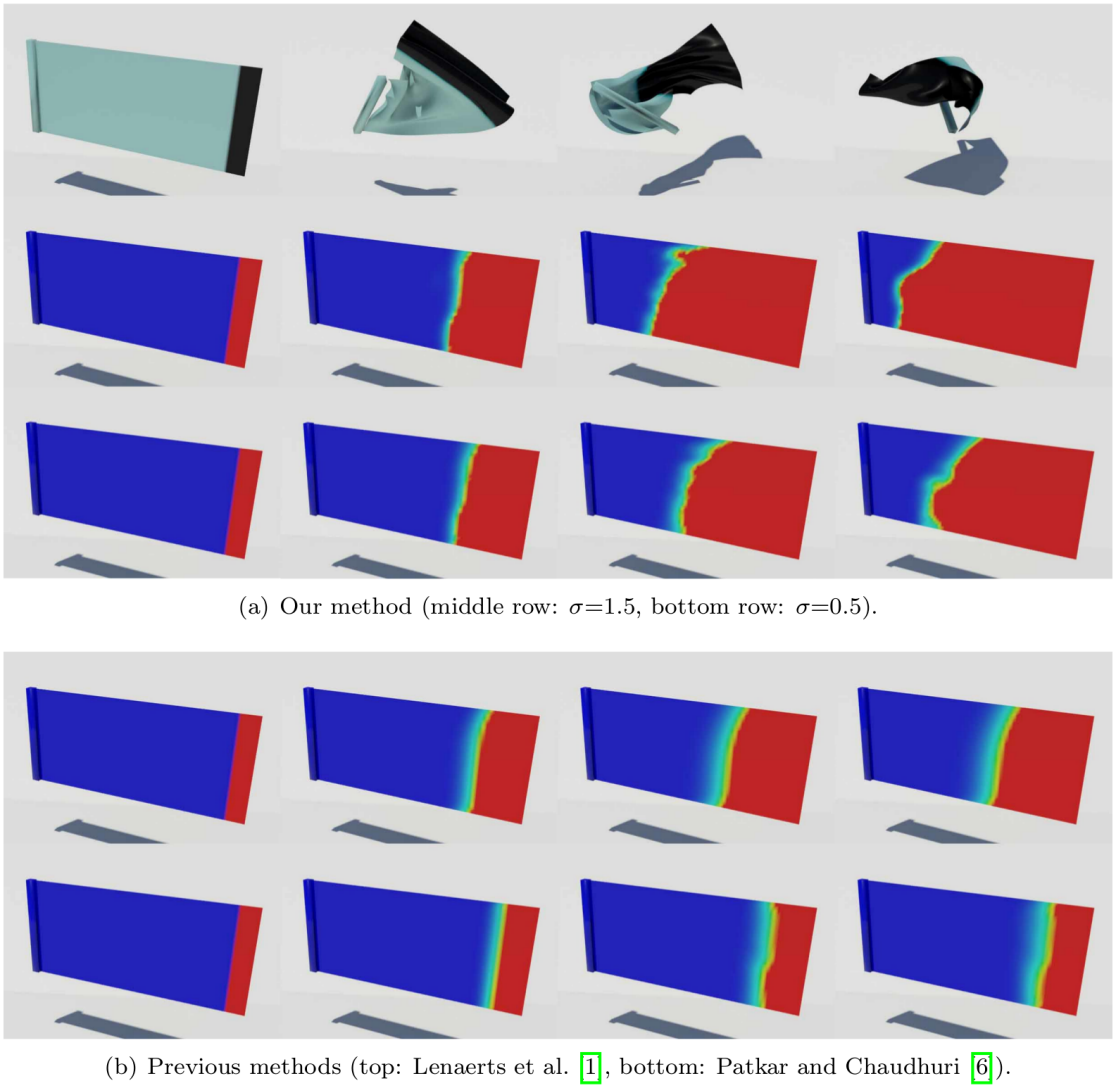
Streaming flag. Diffusion patterns in a streaming flag.

Fig 19 shows that diffusion patterns are represented more clearly for a large deformation. In contrast to the previous methods showing monotonous patterns even for the large deformation of a flag (see Fig 19b), our result shows anisotropic diffusion according to its deformation and it is still maintained when *σ* is adjusted. As mentioned before, *σ* controls the divergence. So just entire extent of diffusion is controlled if we set it to bigger value than zero, and diffusion front is still expressed in detail (see Fig 19a).

## Conclusions and future work

We show how to break the monotony of the diffusion patterns that occur in many scenarios when fluids and solids interact by introducing a divergence-constrained diffusion solver that responds to strain. Our method requires a little more computational cost than previous methods, but it produces simulations that show more details of combustion and water absorption.

Eq (2) is the heat equation, where *α* represents the thermal diffusivity. In general, *α* has a value of 1, but in this paper, this value is changed to control the diffusion rate of heat or water in the solid. Similarly, in the field of computer graphics, the above equations have been applied to various studies to spread water. Among the existing papers, Lenaerts et al. [1] simulated porous flow and wet cloth by applying Darcy’s law and heat diffusion. In addition, Patkar and Chaudhuri [6] proposed a new diffusion model based on solid geometry information and angle of gravity direction to express wetting effects. Both techniques are based on heat diffusion, and our method controls the diffusion rate of water by controlling *α* as well. As shown in Fig 19, the diffusion rate can be controlled by using *σ*. Through several experiments, we confirmed that the use of *σ* rather than *α*, which constantly changes the diffusion rate, better expresses the detail of the diffusion front.

**Fig 20:**
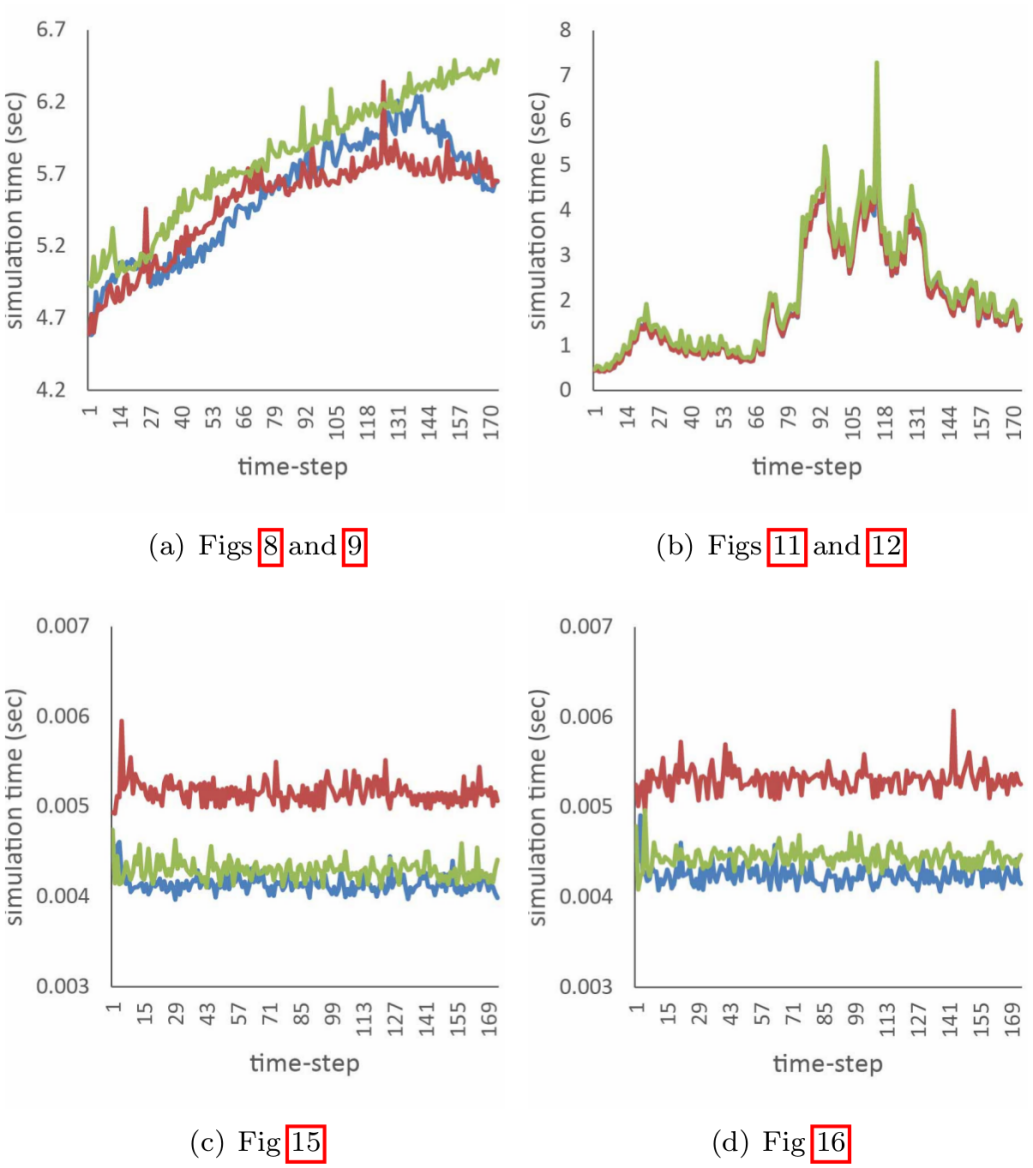
Computational costs of the simulations (red: Lenaerts et al. [1], green: Patkar and Chaudhuri [6], blue: our method).

To enable reproduction of our method by anyone, we have provided the configuration parameters used in comparison with the previous and our schemes in Tables 2 and 3. Fig 20 compares entire simulation times of our method and previous ones. The computational cost of our method is similar to the others because we require only the stages enforcing divergence-constrained calculation of strain. We have experimented with our method in various scenarios and have efficiently expressed the shattering patterns and alive anisotropic patterns in the combustion stage by deformation.

**Table 2:** Simulation parameters with Lenaerts et al. [1] and Patkar and Chaudhuri [6].

Fig	[1] (*k*_*c*_, *α*, *β*, *η*)	[6] ($\alpha ,t_{absorb},k_{d},k_{d}^{\prime },t_{diff}$)
1	15,000, 0.1, 7, 1.0	0.1, 0.002, 0.005, 0.001, 0.424
9	init.: 19,602	–
12	44,016	–
13	10,000	750,000
15, 16	19,607	–
19b	6,448	–

**Table 3:** Simulation parameters with our method.

Fig	Solid_*triangle*_	Water_*particle*_	*σ* (control parameter)
1	init.: 19,602	–	(a): 1.0
4, 6	init.: 19,602	–	–
8	init.: 19,602	–	1.0
9, 10	init.: 19,602	–	–
11	44,016	–	1.0
12	44,016	–	–
13	10,000	750,000	1.0
14	19,607	–	1.0
15, 16	19,607	–	(left): 1.0
17	44,016	–	(a): 1.5, (b): 2.0
19a	6,448	–	(middle): 1.5, (bottom): 0.5

Because our method depends on strain, it would produce isotropic diffusion patterns similar to previous approaches, if it were applied to rigid solids. Our proposed solver satisfies the divergence-constrained diffusion equation globally, so it cannot express local features such as wrinkles. Ashes temporarily appear when clothes is burning, but we do not handle this process. We plan to introduce local features into the diffusion process by explicit detection of ridges and valleys.
